# Blue Rubber Bleb Nevus syndrome: case report from Syria

**DOI:** 10.1093/omcr/omac045

**Published:** 2022-05-23

**Authors:** Joud Haddad, Riham Salloum, Abdullah Omar, Ayham Badran

**Affiliations:** 1 Department of Dermatology and Venereology, Tishreen University, Latakia, Syria; 2 Department of Dermatology and Venereology, Damascus University, Damascus, Syria; 3 Faculty of Medicine, Syrian Private University, Damascus, Syria; 4 Department of Dermatology and Venereology, Damascus Hospital, Damascus, Syria

## Abstract

The rare syndrome of Blue Rubber Bleb Nevus is known to be causing skin vascular lesions in the form of bluish papules, called blue nevi and the movable rubber-like consistency soft tissue masses. The syndrome frequently involves digestive system besides other visceral organs such as liver, lungs, thyroid gland, spleen and nervous system. We present a case of a 36-year-old female with Blue Rubber Bleb Nevus that involved skin and musculoskeletal system.

## INTRODUCTION

The rare BRBNS (Blue Rubber Bleb Nevus syndrome) was first reported by Gascoyen in 1860 [1]. It is characterized by malformations in the venous system of the skin and visceral organs. These malformations could be severe enough to cause bleeding and death. The malformations inhabit the skin in 93% of cases manifesting as button-like bluish papules and the gastrointestinal tract in 76% of cases causing bleeding, iron-deficiency anaemia and further complications such as torsion and intussusception [1]. Symptoms can vary depending on the organ involved. Vascular lesions in the nervous system may cause infarctions and bleeding, while respiratory involvement may include hemoptysis. Only 200 cases of the same syndrome were reported so far. Cancerous lesions have not been reported [2].

Diagnosis is mainly clinically supported by different modalities. Depending on the patient’s presentation and suspicion, the CBC is used to look for anaemia and faecal occult blood could be used to look for bleeding digestive system lesions. Dark urine will necessitate a urine test as bladder lesions could be the cause. Other radiological diagnostics are obtained depending on the specific manifestation, and for instance, musculoskeletal complaints may indicate the need for a simple X-ray or an MRI, while gastrointestinal incidents may require a CT or an endoscopy [1].

Histopathological findings include a vascular network of dilated tortuous blood-filled vessels, lined up with a single layer of endothelial cells, surrounded by a delicate connective tissue [1].

There is no curative treatment. Systemic steroids are used to control intestinal growth. Interferon-alpha is used to treat coagulopathies. Surgical interventions (Surgical excision) or electrocauterization and cryosurgery are only used to excise symptomatic lesions and for cosmetic considerations [3].

## CASE REPORT

A 36-year-old female nurse from a remote rural area in Syria without significant medical or family history presented to the Department of Dermatology of Damascus University with skin complaints of ‘deep blue pimples’ and rubbery consistency masses under her skin in different parts of the body.

As described by the patient, the lesions started appearing right after birth as pigmented papules disseminated on the back and extremities. The papules grew gradually to form nodules and small tumours that bleed easily when exposed to trauma. Nodules also erupted on mucous membranes and the ones on the tongue and tonsils were surgically removed due to dysphagia and discomfort they caused the patient. The lesions kept appearing until the patient reached 20 years of age. In addition to the papules, the patient noticed the appearance of soft masses of different sizes under the skin, some of which was tender.

**Figure 1:**
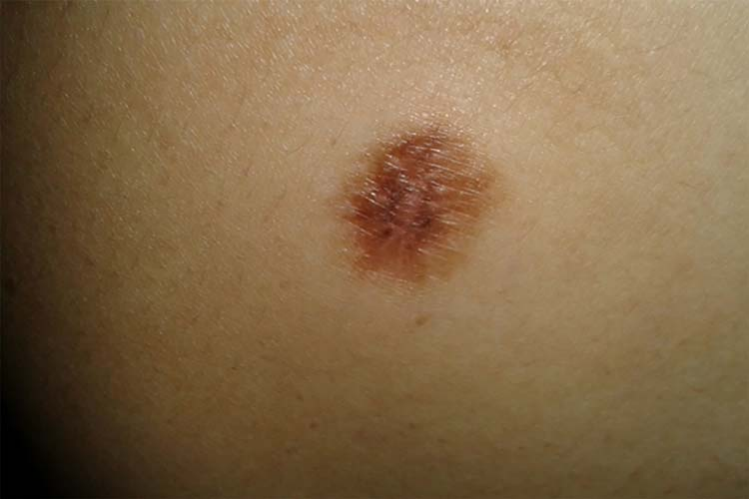
A pigmented brown-blue patch on the back (8 mm × 7 mm).

**Figure 2:**
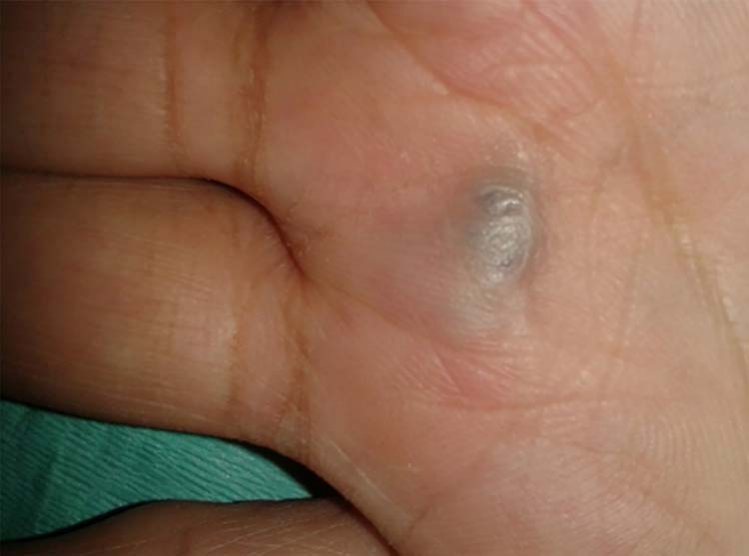
Several pigmented papules on the palms and soles.

**Figure 3:**
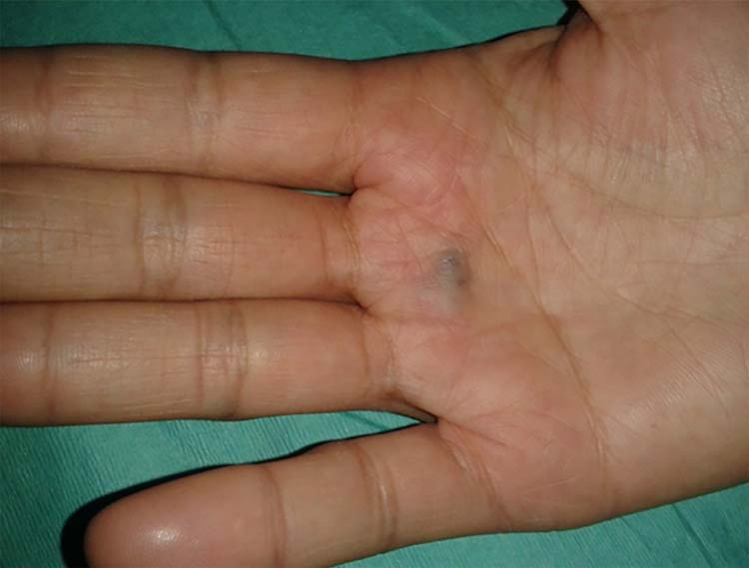
Several pigmented papules on the palms and soles.

**Figure 4:**
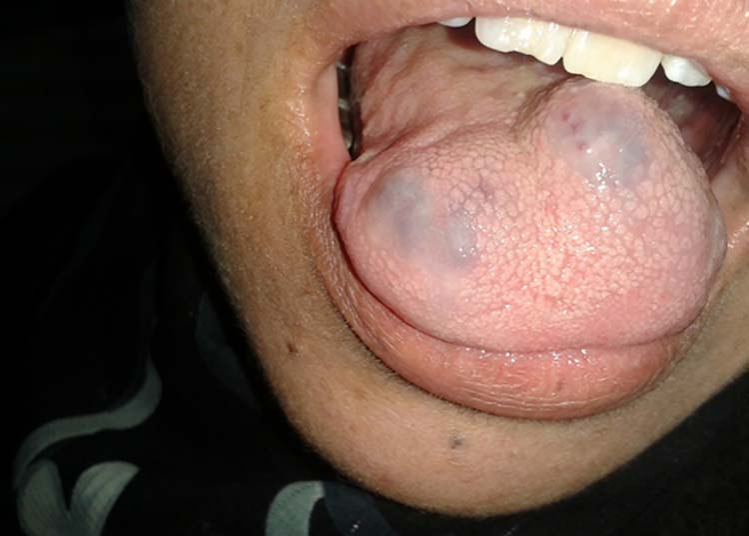
Soft blue rubbery nodules on the tongue.

**Figure 5:**
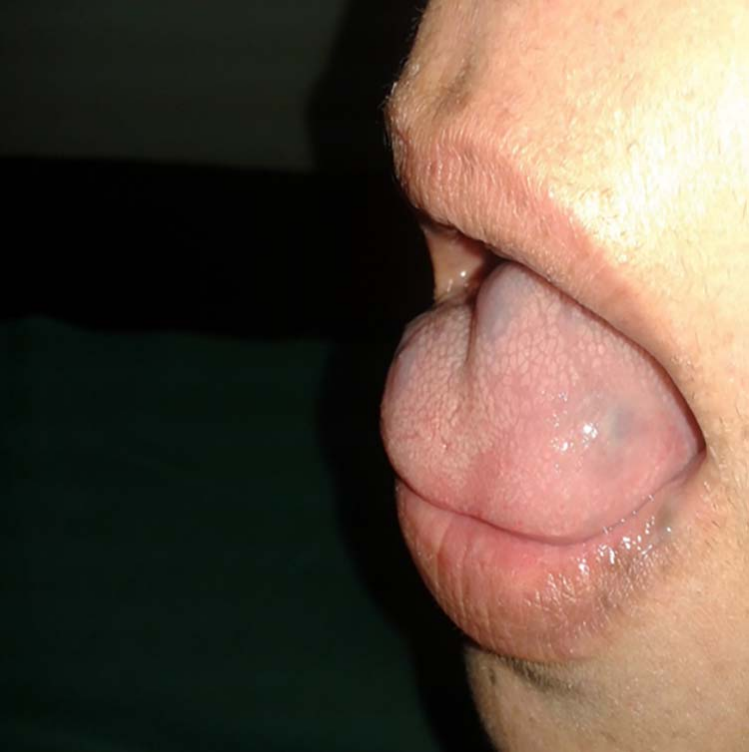
Soft blue rubbery nodules on the tongue.

**Figure 6:**
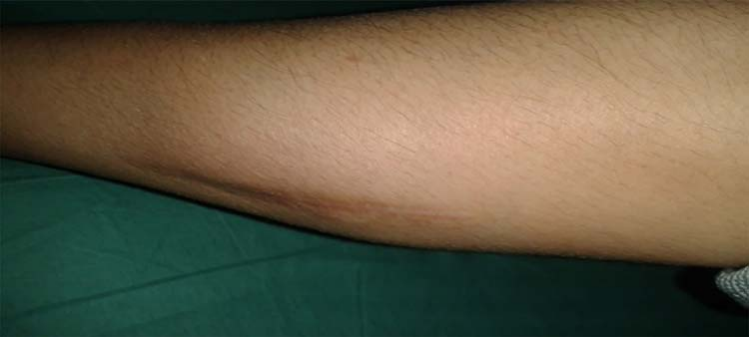
Several mobile soft rubbery subcutaneous tumors on the right forearm (8 cm × 4 cm).

**Figure 7:**
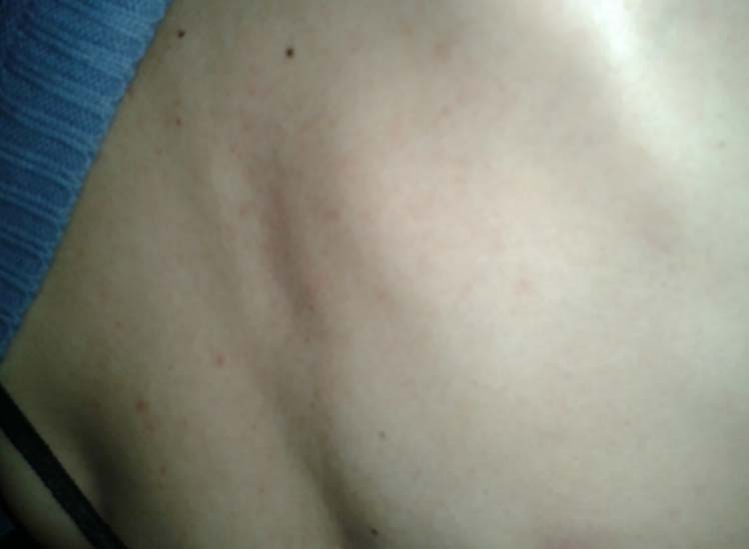
Several mobile soft rubbery subcutaneous tumours on the back (MAX: 8 × 3, MIN: 4 × 3).

Physical examination of the patient revealed a pigmented brown-blue patch on the back (Fig. 1), several pigmented papules on the palms and soles (Figs 2 and 3), soft blue rubbery nodules on the tongue (Figs 4 and 5) in addition to several mobile soft rubbery subcutaneous tumours on the right forearm (Fig. 6) and back (Fig. 7) that were tender to touch and not attached to the overlying skin.

The patient did not suffer from any anaemia that was due to the CBC laboratory examination.

Apart from the scars of previous surgical excisions, the rest of the physical examination was normal including the lymph nodes, hair and nails.

Additionally, the patient reported musculoskeletal complaints of bone and joint pain for which an MRI was ordered only to disclose multiple tumours occupying the muscles of the forearm (Fig. 9).

**Figure 8:**
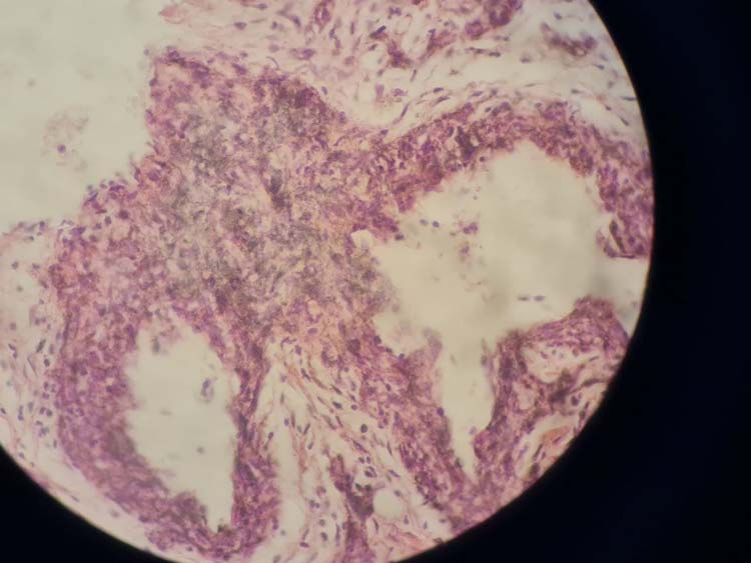
Hematoxylin and eosin slide took from a biopsy from an excided papule.

**Figure 9:**
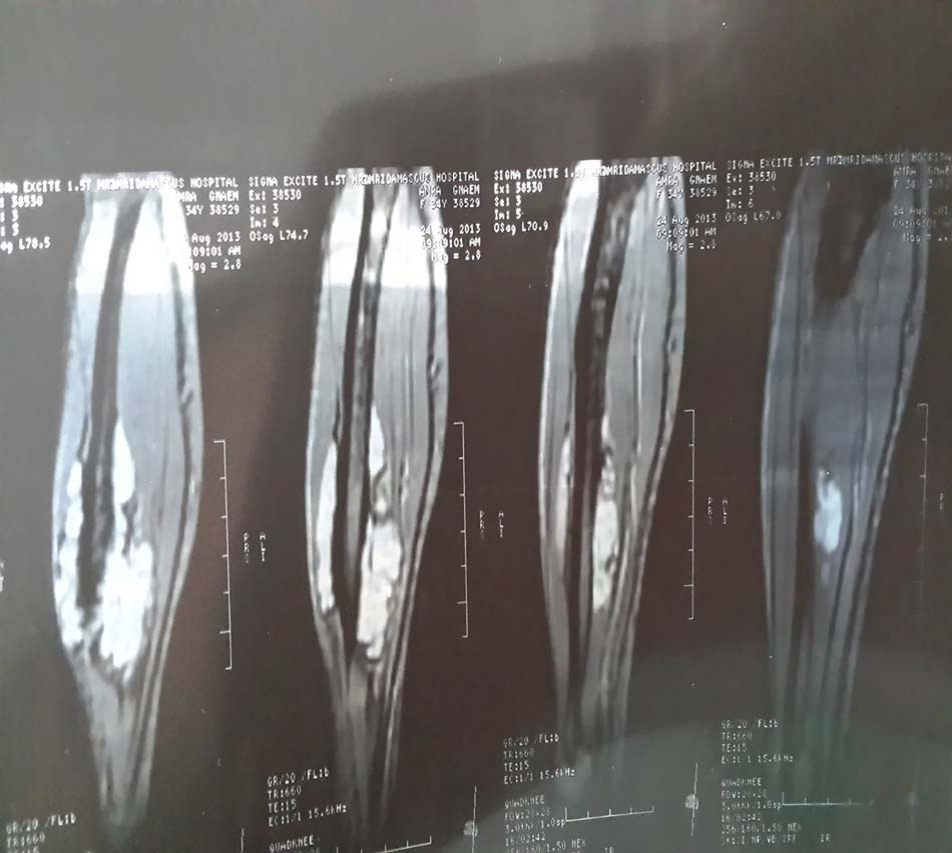
Showing the forearm MRI.

At the age of 6, a biopsy was performed on one of the excised lesions by a dermatologist (Fig. 8). Histological findings described by the pathologist were large dilated vessels in the lower dermis and the subcutaneous tissue accompanied by capillary vascular dilatations in the upper dermis. These findings of a cavernous vascular tumour combined with the overall presentation consider BRBNS, also previously referred to as Bean Syndrome.

The patient was offered cryosurgery or electrocauterization to remove bothersome lesions as she wishes.

## DISCUSSION

To differentiate this diagnosis from similar diseases and syndromes, we compared the specifications of our case with six other entities: Diffuse Neonatal Hemangiomatosis, Familial glomangiomatosis, Maffucci syndrome, Kaposi sarcoma and Klippel–Trenaunay–Weber syndrome [2].

Absent at birth and developing during the first weeks of life, the Diffuse Neonatal Hemangiomatosis is characterized by multifocal tumours up to 20 mm in size that grows rapidly and regresses spontaneously without sequela when confined only to the skin. The tumours could form in the liver, lungs or nervous system, causing symptoms of obstructive jaundice, heart and lung failure, or intracerebral haemorrhage. The mortality rate is high in untreated individuals. Only large symptomatic lesions should be excised. Histopathology reveals endothelial cell hyperplasia in a lobular form, mast cells and a prominent basement membrane. Upon regression, lipofibromatous tissue is seen with mast cells [4]. Our patient started developing skin lesions at birth which did not regress spontaneously, so the diagnosis of Diffuse Neonatal Hemangiomatosis was unlikely.

While being not congenital, Familial Glomangiomatosis manifests with hard (not rubbery) dark blue papules and nodules clustered in groups with less mucosal involvement. The lesions usually start in young children and are less likely to cause symptoms. Some patients report pain episodes elicited by menstruation and pregnancy. The biopsy findings could catch the diagnosis as they show venous malformation of glomus cells surrounding irregular veins [5].

The genetic syndrome of Maffuci generally shows a manifestation at the age of 4–5 years old with cartilage tumours and vascular anomalies including spindle cell endothelial tumours. The nodules are tender and asymmetric, localized more on the distal extremities. The hemangiomas could be superficial or deep. The probability for chondrosarcoma is possible around the age of 40 [6]. Our patient’s age on symptom onset and that she was having central lesions on her trunk and in her oral cavity did not fit the classical picture of Maffuci syndrome.

In contrast to our patient who had several lesions, Klippel–Trenaunay–Weber presents with one large vascular nevus, mostly on the thigh, knee or leg, with overlying lymphatic bullae that could bleed after puberty. The syndrome also has bone and connective tissue hypertrophy with varicose veins that may or may not accompany deep venous anomalies. Doppler ultrasound is used to diagnose this syndrome along with MRI [1].

The nodular type of Kaposi Sarcoma can manifest in older males as lesions mainly on lower extremities and rarely on the face or torso. The lesions can be symmetric with accompanying lymphedema. Hyperkeratotic overlying skin can be seen with a chance of malignancy in the advanced lesions. The biopsy shows bland spindle cells forming slit-like spaces occupied by red blood cells [7].

## CONCLUSION

Our case represents one of the rarest vascular syndromes where only skin, mucous membranes and musculoskeletal systems were involved. The importance of the biopsy is not to be forgotten in combination with the clinical assessment being put in the picture of the whole presentation, such as the age and the progression. Patient education and life-long follow-up are essential to detect probable more dangerous manifestations which might be life-threatening such as gastrointestinal and neurological manifestations.
